# Defining the complementarities between antibodies and haptens to refine our understanding and aid the prediction of a successful binding interaction

**DOI:** 10.1186/s12896-015-0217-x

**Published:** 2015-10-24

**Authors:** Mohammed M. Al Qaraghuli, Soumya Palliyil, Gillian Broadbent, David C. Cullen, Keith A. Charlton, Andrew J. Porter

**Affiliations:** College of Life Sciences and Medicine, University of Aberdeen, Aberdeen, UK; Scotia Biologics Ltd., Foresterhill, Aberdeen, United Kingdom; School of Aerospace, Transport, and Manufacturing, Cranfield University, Cranfield, UK

**Keywords:** Hapten, Phage display, Antibody modeling, Hapten docking, Canonical structure, CDR length, Electrostatic potential

## Abstract

**Background:**

Low molecular weight haptens (<1000 Da) cannot be recognized by the immune system unless conjugated to larger carrier molecules. Antibodies to these exceptionally small antigens can still be generated with exquisite sensitivity. A detailed understanding at the molecular level of this incredible ability of antibodies to recognize haptens, is still limited compared to other antigen classes.

**Methods:**

Different hapten targets with a broad range of structural flexibility and polarity were conjugated to carrier proteins, and utilized in sheep immunization. Three antibody libraries were constructed and used as potential pools to isolate specific antibodies to each target. The isolated antibodies were analysed in term of CDR length, canonical structure, and binding site shape and electrostatic potential.

**Results:**

The simple, chemically naïve structure of squalane (SQA) was recognized with micromolar sensitivity. An increase in structural rigidity of the hydrophobic and cyclic coprostane (COP) did not improve this binding sensitivity beyond the micromolar range, whilst the polar etioporphyrin (POR) was detected with nanomolar sensitivity. Homoserine lactone (HSL) molecules, which combine molecular flexibility and polarity, generated super-sensitive (picomolar) interactions. To better understand this range of antibody-hapten interactions, analyses were extended to examine the binding loop canonical structures and CDR lengths of a series of anti-hapten clones. Analyses of the pre and post- selection (panning of the phage displayed libraries) sequences revealed more conserved sites (123) within the post-selection sequences, when compared to their pre-selection counterparts (28). The strong selection pressure, generated by panning against these haptens resulted in the isolation of antibodies with significant sequence conservation in the FW regions, and suitable binding site cavities, representing only a relatively small subset of the available full repertoire sequence and structural diversity. As part of this process, the important influence of CDR H2 on antigen binding was observed through its direct interaction with individual antigens and indirect impact on the orientation and the pocket shape, when combined with CDRs H3 and L3. The binding pockets also displayed electrostatic surfaces that were complementary to the hydrophobic nature of COP, SQA, and POR, and the negatively charged HSL.

**Conclusions:**

The best binding antibodies have shown improved capacity to recognize these haptens by establishing complementary binding pockets in terms of size, shape, and electrostatic potential.

## Background

The vertebrate immune system is a highly specific defensive barrier to infection that can develop antibodies with the remarkable capability to recognize different antigen classes such as proteins, carbohydrates, and lipids. The antibody molecule contains two light chains and two heavy chains. Every light chain is composed of one variable (V_L_) and one constant (C_L_) domain, and each heavy chain of these antibodies (IgG, IgA, and IgD) folds into four domains: one variable (V_H_), and three constant (C_H1–3_). The V regions of each of these chains comprise three complementarity determining regions (CDRs) separating four more conserved framework (FW) regions [1]. V domains are produced by random rearrangement of germline variable (V_H_), diversity (D_H_), and joining (J_H_) gene segments at the heavy chain loci, and of V_L_ and J_L_ gene segments at the light chain locus [2]. The primary repertoire of antibodies is generated through the assembly of these genes by somatic DNA recombination, and its size depends on the diversity of these genes. During the assembly process, further junctional diversity is introduced at the gene segment junctions by palindromic and nontemplated nucleotides addition through the activity of V(D)J recombinase [3]. Additionally, the primary repertoire diversity is enhanced by combinatorial linkage of heavy and light chains. This second phase of diversification is antigen dependent, occurs in the activated B-cells, and arises from three mechanisms: somatic mutations, gene conversion, and class-switching [4–6].

Despite the potential to generate an almost incalculable variability in final domain sequences and the lengths of the hypervariable loops, these regions have been shown to exhibit a much smaller number of core main chain conformations, which are usually referred to as "canonical structures" [7]. The conformation of a specific canonical structure is determined by the loop length and the presence of specific structural residues at key sites along the domain [8, 9]. Apart from the particularly variable CDR H3 loop, different canonical classes have been assigned to CDR H1 and H2 of the heavy chain [10], and the three CDRs of the kappa and lambda light chains [7, 11, 12]. Although numerous canonical combinations (~300) are theoretically possible, domination of relatively few combinations is typically observed for antibodies specific for protein, carbohydrate, or hapten antigens [13, 14].

Haptens are ubiquitously distributed in our environment as pollutants, and widely utilized as pharmaceuticals, hormones, and antibiotics. These small molecular weight molecules (below 1000 Da) cannot be efficiently recognized by the mammalian immune system unless conjugated to larger immunogenic carrier molecules [15]. “Tricking” the immune system in this way has allowed anti-hapten binders with exquisite specificity and sensitivity to be successfully generated from a number of sources [16, 17]. Antibody based immunosorbent assays have been developed as efficient and inexpensive tools for hapten detection [18]. Antibodies developed against various antigen classes have been shown to display different binding surfaces that tend to accommodate the shape of the target antigen [19]. This phenomenon can be summarized as deep binding pockets for anti-hapten antibodies, and binding grooves for linear peptide antibodies, or antibodies that bind proteins possessing relatively flat binding sites [20]. The development of cavity-like binding sites that recognize haptens has been reported for a number of species including: human [21], mouse [22], llama [23], and rabbit [24].

Several studies have attempted to analyze in greater detail the relationship between antigen specificity and binding site structure. Each study has tended to focus on the application, in isolation, of a number of different approaches including: somatic hypermutation [25], continuum electrostatic methods [26], CDR length and sequence composition [27], and conformational changes on binding [28]. However, no previous research has tried to use these strategies collectively to analyze antibodies developed against different hapten classes. In fact, the main focus of research within the anti-hapten antibody field has been typically limited to the characterization of anti-hapten binding sensitivity and cross-reactivity in relation to their targets; rather than considering how this remarkable ability of antibodies to recognize a small, but diverse class of molecules is achieved at the molecular level. Consequently, we describe the development and characterization of panels of antibody binders to four very different haptens: SQA, POR, COP, and HSL, all derived from a series of immunized sheep phage display libraries. SQA represented the minimum signature in terms of its simplicity, hydrophobicity, and flexibility of chemical structure. An increase in structural rigidity was offered through the selection of the haptens COP and POR. However, the former lacks the presence of any polar group, when compared to POR, which possess four nitrogen atoms within its core. Finally, HSL molecules were chosen as they combine molecular flexibility with the presence of potential polar groups. Here our characterization approach has focused on the antibody-hapten interaction and the complementarities of the binding counterparts in terms of their shape, size, and electrostatic energy. Furthermore, these factors were correlated with the canonical structures, CDR lengths, amino acid distribution, and antigen sensitivities of these anti-hapten binders to generate detailed and predicted models of anti-hapten antibody binding sites.

## Methods

### Sheep immunization and sera characterisation

Three conjugates were prepared (Cranfield University) following standard protocols [18], and characterized by the Aberdeen Proteomics Facility using a MALDI-MS system. In collaboration with Ig-Innovations Ltd (Llandysul, UK), the sheep were immunized with the desired hapten-conjugates (Table 1). Animals were immunized with 100 μg/ml initial concentration, and four subsequent 50 μg/ml boosts, which were administered subcutaneously atsix sites. Freund's adjuvant was used in the immunization process, as a complete form (Sigma, F5881) in the initial dose, and as an incomplete form (Sigma, F5506) in the four successive boosts. Bleeds were taken from the jugular vein two weeks after each boost. All animal work/procedures carried by Ig Innovations Ltd, are performed under a rigorous Home Office (Scientific Procedures) Licence. The project Licence covers antibody production in large animals and describes the various techniques and procedures that were used as part of this study. Lymphocytes were isolated from the blood using Histopaque®-1077 (Sigma-Aldrich, 10771) and Accuspin tubes (Sigma-Aldrich A2055), and the isolated lymphocytes were stored in RNA stabilization buffer (Qiagen, 76104). Sera characterization was performed using binding and competition ELISA following protocols that were described previously [29]. The ELISA plates were coated with hapten-carrier protein conjugates at 1 μg/ml, and donkey anti-sheep (whole antibody) HRP antibody (Sigma, A3415) was utilised as the secondary antibody to generate a binding signal.

### Phage display library construction

Phage display library construction followed standard protocols [16, 30]. Briefly, RNA was isolated from whole blood lymphocytes using RNeasy® Midi Kit: (QIAGEN: 75142). cDNA was then synthesized by PCR using three sheep forward primers (OvCHFOR: 5’-GAC TTT CGG GGC TGT GGT GGA GGC-3’; OvCλFOR: 5’-A CAG GGT GAC CGA GGG TGC GGA CTT GG-3’; OvCκFOR: 5’-GA TGG TTT GAA GAG GGA GAC GGA TGG CTG AGC-3’). The PCR reaction included 25 μl of DNase treated RNA, 3 μl of 25 pmol forward primers, 8 μl of 5x first strand buffer, 4 μl of 0.1 M DTT, 1 μl of 10 mM dNTPs mixture, and 1 μl (200 U) SuperScript® III Reverse Transcriptase (Invitrogen, 18080044).

Sequence amplification rescuing of the heavy and light chains was conducted by PCR utilizing sheep gene-specific primers [30]. PCR reactions comprising 1 μl (25 pmol) each of OvVH_(1–4)_ BACK primers and combination of OvJH_(1–4)_ FOR primers, 1 μl cDNA, 25 μl 2x HF Phusion® mix (NEB, 531 L), and 22 μl sterile water. The λ and k light chains were amplified as above but using (OvVλ_(1–5)_ BACK/OvJλ_(1–2)_ FOR) or (OvVk_(1–2)_ BACK/OvJk_(1–3)_ FOR) primer combinations. The amplified heavy and light chains were digested with *Asc*I (NEB, R0558) and *Mlu*I (NEB, R0198S) restriction enzymes, respectively. The complementary sites created by digestions with *Asc*I and *Mlu*I permitted ligation of the heavy and light chains and the formation of a 15 amino acids linker (EGKSSGASGESKVDD) between and joining these domains. The linked DNA material was amplified by pull-through PCR using OvVH_(1–4)_ BACKSfi primer and OvJλ_(1–2)_ FORNot or OvJk_(1–3)_ FORNot primers. The linked VH and VL (λ or k) DNA (~800 bp) was purified and digested overnight at 37 °C with (10 U/μg DNA) *Nco*I (NEB: R0193S) and *Not*I (NEB: R0189L) restriction enzymes. The digested DNA was then purified and ligated (T4 DNA ligase; NEB, M0202L) into a pHEN2 phagemid vector. This step was followed by ethanol precipitation of the ligated DNA, and transformation of the vector into electrocompetent *E.coli* TG1 cells (Lucigen, 60502) using an Electroporator (Eppendorf, 2510).

### Library panning and screening

Phage display library selections were performed as detailed previously [17]. In brief, MaxiSorp tubes (Nunc, 444474) were coated with the desired antigen-conjugates, and incubated overnight at 4 °C. The tubes were then washed and phage particles (~1 × 10^12^) added in 4 ml 2 % Marvel-PBS. The bound phage were eluted by the addition of 1 ml of 100 mM triethylamine (Sigma-Aldrich: T0886) for 10 min, then neutralized by the addition of 0.5 ml of 1 M Tris Buffer (pH 7.4). The eluted phage were used to infect *E.coli* (TG1) cells at their exponential growth phase for 30 min in a water bath, and for another 30 min at 37 °C in a shaking (250 rpm) incubator. The cells were then plated on TYE agar containing 1 % glucose and 100 μg/ml ampicillin, and incubated overnight at 30 °C, before being colony scraped and stored at −80 °C. To rescue the library, M13K07 helper phage (GE Health care, 27-1524-01) were used to infect the library and inoculated into the culture at 1:20 ratio (cell:phage). Phage clones were isolated and characterized by ELISA according to established protocols [31].

### Expression and purification of scAb proteins

The plasmid DNA of the anti-hapten scFv were digested with *Nco*I and *Not*I restriction enzymes, and cloned into a similarly digested soluble expression vector pIMS147 [32]. The ligated product was transformed into *E. coli* (XL1-Blue) cells (Agilent Technologies, 200228) by electroporation. Expressions of scAbs in transformed *E. coli* XL-1 Blue bacterial cells were carried out in Terrific Broth according to standard protocols [33]. The expressed scAbs were purified *via* the hexa-histidine tag using immobilized metal ion chelate affinity chromatography (IMAC). The sensitivity of expressed scAb proteins for their target antigen was examined using a scAb binding ELISA [29] and/or an indirect competition ELISA [34]. All ELISA plates were coated with hapten-carrier protein conjugates at 1 μg/ml.

### Determination of CDR length, canonical structures, and amino acid distribution

The CDRs lengths of the heavy and light chains were defined throughout this article following the well-established Kabat numbering system. To analyze amino acid distribution and conservation throughout the entire antibody, amino acids were classified into seven groups using a Microsoft EXCEL visual basic macro sheets [35]. These sheets were downloaded from the AAAAA server [36]. Here again, each amino acid, within the analyzed sequences, was numbered according to the Kabat scheme [1, 37]. In addition, the canonical classifications of the loops were determined according to a Chothia SDR template [7]. These numbering and classification steps were further aided by reference to Dr. Andrew C.R. Martin's Group website (http://www.bioinf.org.uk/abs/chothia.html).

### Antibody modelling and electrostatic potential measurements

The variable domains of anti-hapten antibodies were modelled using the project mode in the SWISS-MODEL website [38]. The heavy and light chains templates were identified using the SWISS-MODEL workspace template identification portal. Templates with the highest sequence identities, and similar canonical classes, were then selected. The homology models of the heavy and light chains were generated by the SWISS-MODEL workspace automated modelling portal with appropriate specification for each selected template. The generated model qualities were then examined following standard protocols [39]. Finally, the heavy and light chains were linked by Swiss-PdbViewer 4.0.1, and visualized by PyMOL (academic version 1.3). Electrostatic energy of the selected antibodies was calculated using Adaptive Poisson-Boltzmann Solver (APBS) Version 0.5.1 in the Python Molecule Viewer (PMV) Version 1.5.6. The produced energy was mapped to the surface with medium surface quality and at a 1 Å distance. The map colour was coded as white: 0 kT/e, Blue: 10.2 kT/e, Red: −10.2 kT/e.

### Docking analysis (AutoDock vina)

Automated docking was used to predict suitable binding orientations and conformations of the various haptens positioned at/within their corresponding binding sites. The antibody-antigen docking analyses were conducted using the molecular docking and visual screening program AutoDock vina [40]. Polar hydrogen atoms were added, and the generated models were saved as pdbqt files. The ligand (free antigen) rotatable bonds were examined and saved in similar pdbqt format. A potential option within AutoDock vina is the ability to determine the docking site of the antibody by setting the dimensions of the docking grid box. This was achieved by setting the x, y, and z axes of the grid box to cover the binding sites of the antibody. The docking process was achieved using the command prompt within Windows 8. The command script included [>cd "Desktop\(file name)”], [ >\Program Files (x86)\The Scripps Research Institute\Vina\vina.exe" –help], [>\Program Files (x86)\The Scripps Research Institute\Vina\vina.exe" --config conf.txt --log log.txt]. Upon completion of the docking process, the models were exported, viewed and analyzed by PyMOL (academic version 1.3).

## Results

### Library construction, bio-panning, and characterization

For each immunized sheep, lymphocytes were used as the genetic source for scFv. Sheep sera samples were routinely examined during the immunization process, and the selection of material for library construction made based on the best measured responses. Sequences of the different antibody fragment libraries confirmed a full diversity within the CDR loops. Each of the libraries was considered highly diverse, containing at least 10^7^-10^8^ unique clones. The panning strategies employed to select phage-binders included a series of steps to encourage the enrichment and selection of the more sensitive and specific clones [31]. Whilst this is not an exact science, care was taken where possible to follow a similar selection strategy for each hapten class (increasing stringency though reduction in antigen concentration and the swapping of protein conjugates to minimize the selection of carrier protein specific clones etc.). The selection process was stopped when clones of the same sequence began to appear at a level of great than 30 % of positive clones analyzed. Typically this was after 3–4 rounds of selection and bio-panning. Binding and competition ELISAs of monoclonal phage were used to identify unique clonal panels specific for SQA, POR, HSL, or COP (Table 1). The isolated scFv phage clones were converted into a scAb (single chain antibody) format, and the sensitivities of purified soluble antibody fragments to free haptens were determined by competition ELISA (Table 2). The “naïve” or simple chemical structure of SQA was recognized with only micromolar sensitivity (IC_50_). An increase in the structural rigidity of the hydrophobic and poly-cyclic second antigen COP did not really improve this moderate IC_50_ sensitivity beyond the micromolar range. However, clone POR B11 was able to detect the rigid but polar POR with an IC_50_ value of 270 nM. The strongest molecular recognitions were seen for HSL molecules, which comprise a hydrophobic tail attached to a hydrophilic lactone ring. Several anti-HSL antibodies displayed super-sensitive interactions with clone HSL 1 having an IC_50_ in the picomolar range (500 pM).

**Table 1:** Summary of the sequence analysis strategy

	Immunised antigens	Library size	Sheep type	Number of pre-selection sequences		Number of post-selection clones
				H	L (λ)	
Library 1	SQA and POR	4.83 x 10^8^	Cheviot cross	110	80	SQA (6) POR (3)
Library 2	COP	1.26 x 10^8^	Welsh Suffolk cross	77	30	COP (8)
Library 3	3-OXO-C12-HSL, 3-OH-C12-HSL, and N-acyl-C12-HSL	4.1 x 10^7^	Welsh Suffolk cross	196	95	HSL (6)

The analyzed sequences were obtained from three libraries. The pre and post-selection sequences included heavy (H) and lambda light (λ) chains. Pre-selection clones were randomly selected sequences during library construction process

**Table 2:** Binding sensitivities of the selected antibodies

Target	Molecular weight (Da)	Structure	Isolated clones	IC_50_ (μM)
3-OXO-C12-HSL	297.39		1	0.0005
			2	0.0015
			3	0.0025
			4	0.0005
			5	0.0025
			6	0.0004
SQA	422.81		A5	2
			B3	7.5
			F1	#
			F9	4.5
			E7	#
			E10	2.2
POR	478.67		D11	1
			A7	2
			B11	0.27
COP	372.67		E12	30
			F12	15
			H3	60
			28	#
			A5	#
			A8	#
			F3	#
			G12	#

Summary of the binding sensitivities of the analyzed clones together with the chemical structures of their corresponding antigens. #: IC_50_ values were not successfully detected

### Sequences analysis

Sequences were compared for clones from the pre and post-selection (panning) and are summarized in Table 1. Sequences were analyzed by examining canonical structures, CDR lengths, and amino acid distribution.

#### CDR length and canonical structures

CDR lengths determination of each individual library has revealed clear conservation in CDRs H1, H2, and L2 (Table 3). In addition, there was moderate diversity in the lengths of CDR L1, and high variability in CDRs H3 and L3. The length of CDRs H1, H2, and L2 were identical for all the post-selection clones isolated from the three libraries, irrespective of binding specificity. In contrast, CDR H3 and L3 (post-selection) lengths could be clustered into two groups: (i) antibodies that were developed against COP (library two) having generally shorter CDR H3 and L3 when compared to (ii) the antibodies developed against SQA, POR, and HSL (from libraries one and three, respectively). In general terms, the selection process has introduced bias into the CDR lengths recovered with CDRs H3, L1, and L3 having lengths in post-selection clones that were represented at low frequency in the pre-selection repertoires.

**Table 3:** CDR length distribution of the pre and post-selection sequences

A	CDR length (percentage of representation within the pre-selection sequences)					
	CDR H1	CDR H2	CDR H3	CDR L1	CDR L2	CDR L3
Library 1	5 (100)	16 (97)	14 (14.5)	14 (65)	7 (100)	10 (62.5)
		18 (2)	13 (11.8)	11 (20)		11 (28.8)
			12 (10)	13 (14)		9 (2.5)
			11 (8.18)			
Library 2	5 (96.1)	16 (98.7)	11 (18)	14 (67)	7 (100)	10 (56.7)
	6 (2.6)	15 (1.3)	14 (16)	13 (20)		11 (30)
	7 (1.3)		13 (12)	11 (13.3)		12 (13.3)
			9 (1.3)			9 (0)
Library 3	5 (100)	16 (100)	13 (16)	14 (52.6)	7 (100)	10 (67.4)
			14 (15)	13 (24.2)		11 (21.1)
			15 (13)	11 (20)		12 (7.4)
			12 (8.7)			
B	CDR length (percentage of representation within the post-selection clones)					
	CDR H1	CDR H2	CDR H3	CDR L1	CDR L2	CDR L3
Library 1	5 (100)	16 (100)	12 (89)	14 (89)	7 (100)	11 (89)
			11 (11)	11 (11)		10 (11)
Library 2	5 (100)	16 (100)	11 (50)	13 (75)	7 (100)	9 (50)
			9 (25)	14 (25)		10 (50)
			13 (25)			
Library 3	5 (100)	16 (100)	12 (100)	13 (100)	7 (100)	11 (100)

The most prevalent CDRs lengths (number of amino acids) within each library in (A) Pre-selection clones and (B) Post-selection clones. The percentage of each specific CDR length within (A) the entire library population or (B) post-selection clones is in parenthesis

Canonical classification was performed utilizing a strict Chothia SDR template, on all the CDRs except CDR H3. The pre-selection sequences from all three libraries comprised heavy chain sequences dominated by a 1–1 canonical combination for CDR's H1 and H2 (Table 4). Typically, these classes corresponded to CDR lengths of 10 (CDR H1) and 9 (CDR H2) amino acids. There was moderate but not unexpected variability in the classification of lambda light chains within pre-selection sequences. A significant contribution to the overall library variability was from a canonical class combination 6-1-X for CDRs L1-L2-L3. CDR L1 class 6 represents a 14 amino acid loop length, while class 1 (7 amino acids loop) is the only identified group for CDR L2 in the literature [7, 41]. Class X is used here to indicate that no canonical class has been reported previously with a similar loop length. The three sampled antibody libraries were analyzed statistically using a chi square goodness of fit test (*X*^*2*^) (IBM SPSS 21) to evaluate whether the canonical class representation was equal within each antibody library. However, the null hypothesis was rejected (p value ≤ 0.001), and therefore, there was domination of specific canonical classes within each library. It is impossible to conclude whether this bias was present as a result of the different immunizations or as an artifact from the library cloning process. Clones (post-biopanning) included only lambda light chains, with a clear antigen specific canonical combination bias seen for each target. In contrast, the heavy chains CDRs H1-H2 were all classified as 1–1 (Table 4); a canonical class combination that dominated all the three libraries’ pre-selection sequences. The post-selection lambda chains were from canonical classes of CDRs L1 and L3 that were present in low abundance within the pre-selection sequences, and reflected the CDR length trends described previously. CDR L1 of the highly sensitive post-selection clones was grouped within class 5 or 6. These sensitive clones included CDR H3 with 9 (class 4) or 11 (class 5) amino acids. In addition, CDR L3 class X was evident in post-selection clones that have not shown high binding sensitivities, like SQA (E7) and COP clones (A5, A8, F3, and G12).

**Table 4:** Canonical combinations of the pre and post-selection sequences

A	Canonical combination (percentage of representation within the pre-selection sequences)	
	CDRs H1-H2	CDRs L1-L2-L3
Library 1	1-1 (97.3)	6-1-X (51.3)
	2-4 (1)	2-1-5 (11.2)
	1-4 (1)	6-1-5 (11.2)
		2-1-X (10)
Library 2	1-1 (94.8)	6-1-X (46.7)
	2-1 (2.6)	6-1-5 (20)
	3-1 (1.3)	5-1-X (16.6)
		5-1-4 (−)
Library 3	1-1 (100)	6-1-X (45.3)
		5-1-X (14.7)
		2-1-X (17.7)
		5-1-5 (5.26)
B	Canonical combination (percentage of representation within the post-selection clones)	
	CDRs H1-H2	CDRs L1-L2-L3
Library 1	1-1 (100)	6-1-5 (88.9)
		2-1-X (11.1)
Library 2	1-1 (100)	6-1-X (25)
		5-1-4 (50)
		5-1-X (25)
Library 3	1-1 (100)	5-1-5 (100)

The canonical classes were determined using Strict Chothia SDR template [7, 9, 53, 63]. Data presented represent the most prevalent canonical combinations within each library in (A) Pre-selection clones and (B) Post-selection clones. X: indicates no currently recognized canonical class with the same loop length. The percentage of specific canonical combination within the entire library population of the analyzed pre (A) and post (B)-selection sequences are in parenthesis

#### Amino acid distribution

**Fig. 1:**
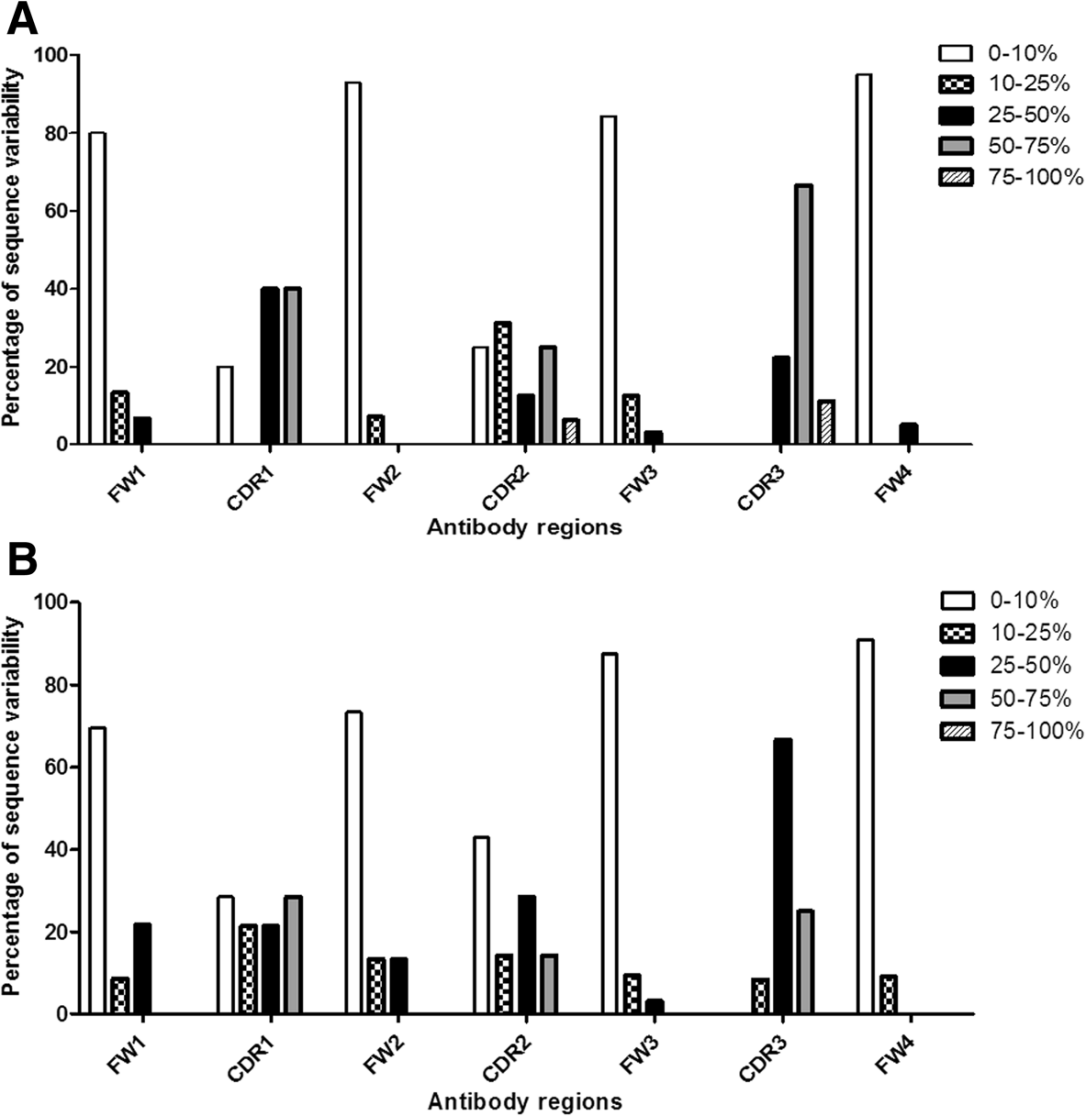
Amino acid variability within various regions of the isolated sequences. Variability within **a** VH, **b** VL regions of the pre-selection sequences. Diversity at each amino acid position was classified into five groups. The total CDR or FW region variability was determined as a percentage of the overall diversity of positions within the selected regions (CDR or FW)

Analyses of the pre-selection sequences confirmed the high level of amino acid site conservation in the FW regions and remarkable variability within the CDR loops (Fig. 1). The majority (~80 %) of the FW positions were well conserved (variability <10 %). Comparison between the sheep heavy and light chain's FW regions indicated that the heavy chain FW positions are 30 % more conserved than the light chain. As anticipated, the most variable region was CDR H3, of which >80 % of the positions showed >50 % variability. The most diverse positions within the heavy chains were H32-33 (CDR H1) and H50-56 (CDR H2). Positions L28-32 (CDR L1) and L50-53 (CDR L2) were highly diverse within the light chains.

In sharp contrast to the broad repertoire diversity seen for the pre-selection clones only a relatively small number (3 to 8) different hapten binders were present (often repeated several times) at the end of each antigen-specific, bio-panning (Table 2). This extreme contraction of repertoire diversity is independent of the hapten type and is further reflected in an extreme narrowing of the canonical structures represented within the hapten binding clones (Tables 4 and 5). It must be concluded, therefore, that only a tiny percentage of the original and highly diverse sheep repertoires, even following bias through immunization and boosts, have a paratope-shape that is pre-disposed to bind haptens, as a class. All post-selection sequences were collectively “searched” for signs of possible hapten-driven similarities or differences. The number of conserved positions were significantly higher in post-selection sequences (123 positions) when compared to their pre-selection counterparts (28 positions) (Table 5). In addition, FW3 of the heavy and light chains contains the highest levels of conservation, possibly identifying the importance of this region in orientating CDRs 2 and 3 required to form a pocket for recognizing haptens. Not unexpectedly, the CDR regions themselves contain a significantly lower level of conservation, as each of these antibody panels was selected against different hapten antigens. Taken together, the concentration of site conservation within the FW regions, but not in the CDRs, appears to suggest an important structural role for the FW regions in enabling CDRs to be displayed in the required orientation to accommodate haptens without compromising antigen affinity.

**Table 5:** Comparison of the conserved amino acid positions following antigen selection

	Pre-selection		Post-selection	
	Heavy	Light (λ)	Heavy	Light (λ)
FW1	3 (19,14, 20)	7 (3, 10, 11, 15, 19, 21, 23)	21 (2–15, 17–22, 25–28)	13 (3–7, 9, 11, 15, 16, 20–23)
FW2	3 (36, 45, 48)	2 (35, 47)	9 (36–43, 45)	6 (35, 40, 44, 45, 47, 48)
FW3	4 (82, 82c, 90, 92)	6 ( 61, 67, 73, 75, 82, 86)	24 (66, 67, 70–72, 74, 76-82c, 86–92)	23 (57–62, 64, 66, 70, 72–75, 77–79, 81–84, 86–88)
FW4	0	3 (99, 102, 104)	10 (103, 104, 106–113)	10 (98, 99, 101–108)
Total (FWs)	10	18	62	51
CDR1	0	0	1 (34)	4 (24, 25, 26, 33)
CDR2	0	0	2 (51, 63)	3 (54, 55, 56)
CDR3	0	0	1 (101)	0
Total (CDRs)	0	0	3	7
Total (chain)	10	18	65	58

Analysis was conducted on the pre and post-selection sequences. A conserved site was defined as: a position (Kabat numbering [37]) in the post-selection clones where an amino acid remained unchanged or had similar bio-physical characteristics to the original pre-selection residues

### Surface analysis of post-selection clones

**Fig. 2:**
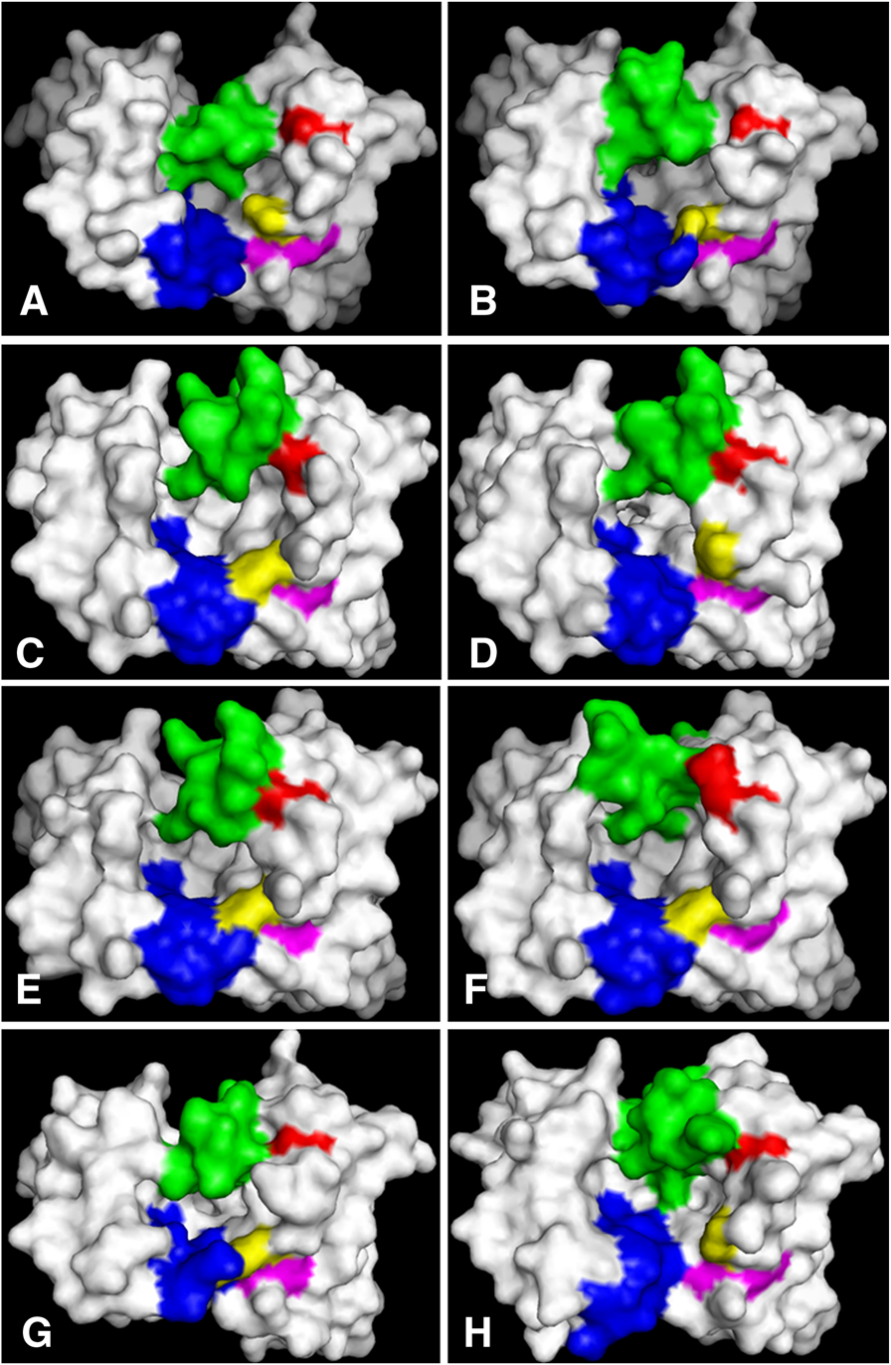
The effect of amino acids within CDR H2 on the predicted positioning of CDRs H3 and L3. Amino acid positions H53 (red), H58 (yellow), and H59 (magentas) in clones **a** HSL1, **b** HSL2, **c** SQA A5, **d** SQA B3, **e** POR B11, **f** POR A7, **g** COP H3, and **h** COP A8. Position H53 has a profound effect on the orientation of CDR H3 (green), whilst positions H58 and H59 influence CDR L3 (blue) orientation. These homology images of the post-selection clones were constructed utilizing SWISS-MODEL workplace. The structures were viewed by PyMOL 1.3 (academic version)

**Fig. 3:**
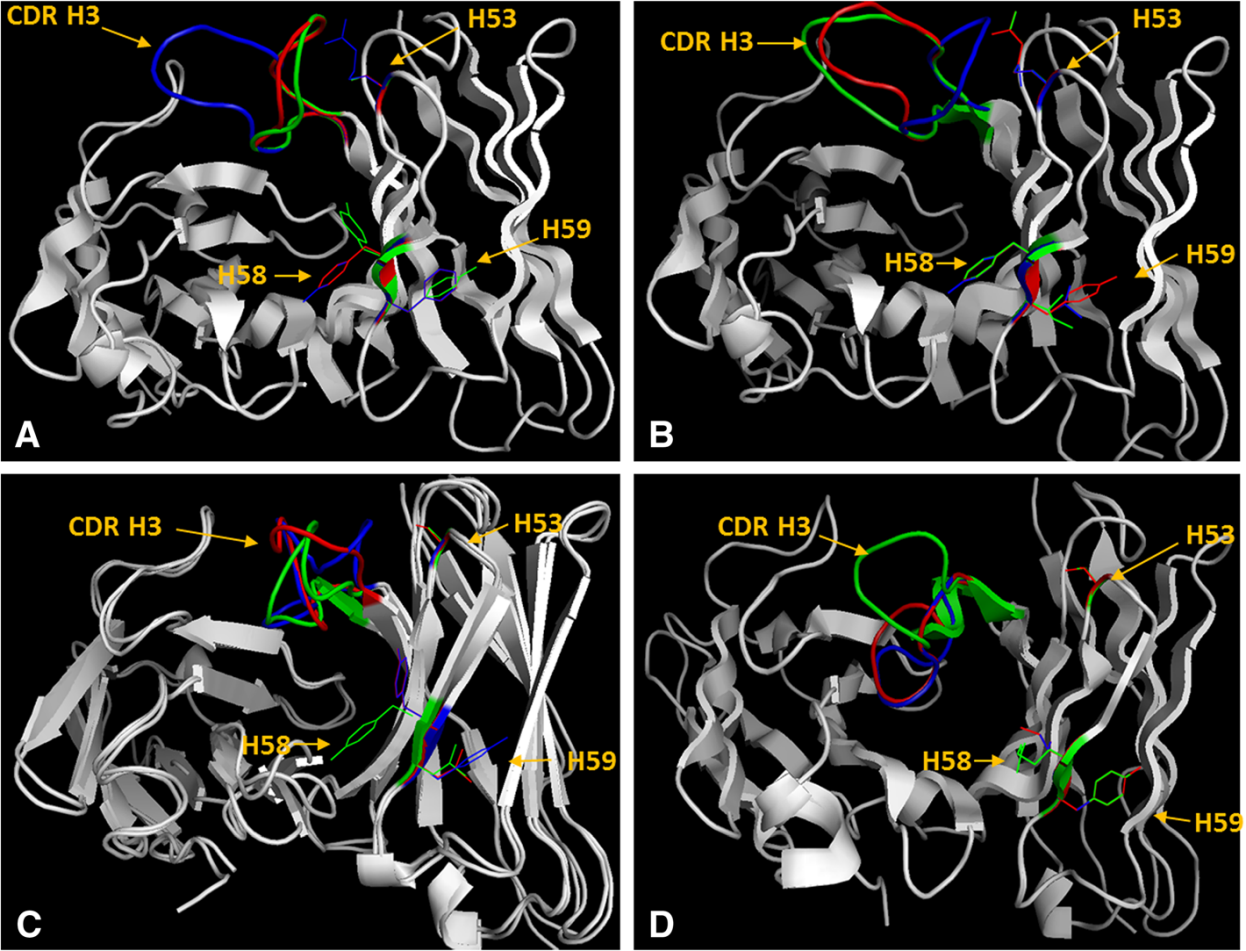
Side-chains orientations of potential amino acids within CDR H2. Site-specific interactions of amino acid positions H53, H58, and H59. **a** SQA A5 (red), SQA B3 (green), and SQA F1 (blue). **b** POR A7 (red), POR D11 (green), and POR B11 (blue). **c** COP E12 (red), COP H3 (green), and COP A8 (blue). **d** HSL 1 (red), HSL 2 (green), and HSL 4 (blue). These homology images of the post-selection clones were constructed utilizing SWISS-MODEL workplace. The structures were viewed by PyMOL 1.3 (academic version)

**Fig. 4:**
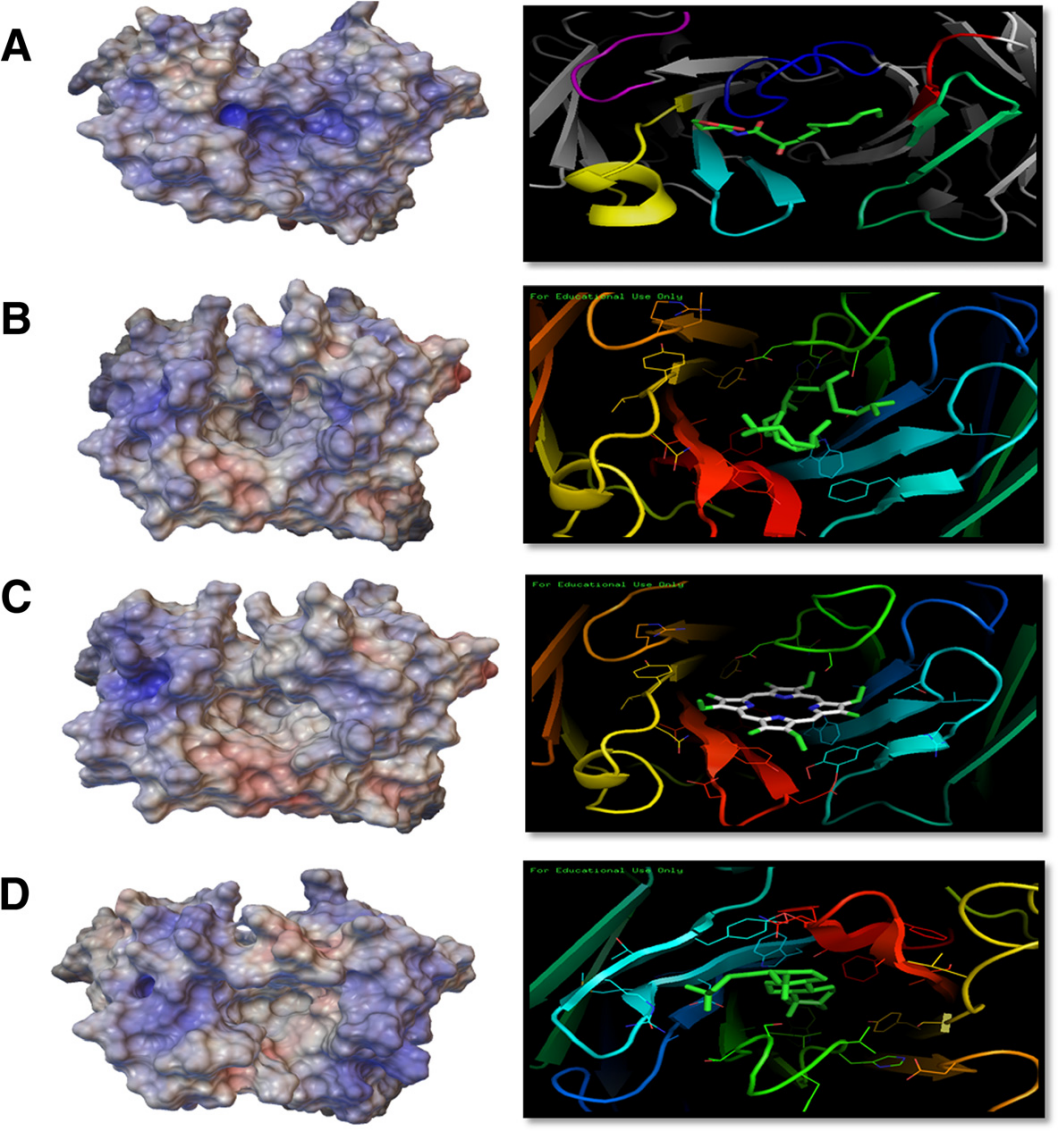
Structural, docking, and surface-mapped electrostatic potential of antibodies. The homology structures of the post-selection clones were determined by SWISS-MODEL workplace for clones **a** HSL1, **b** SQA A5, **c** POR B11, **d** COP H3. Docking analyses were conducted with AutoDock Vina 1.5.6. The structures were viewed by PyMOL 1.3 (academic version). Measurements of electrostatic energies were calculated using Python Molecule Viewer (PMV) Version 1.5.6. The produced energy was mapped with medium surface quality and at a 1 Å distance from the surface. The map color was coded as white: 0 kT/e, Blue: 10.2 kT/e, Red: −10.2 kT/e

Homology models of post-selection clones were constructed utilizing SWISS-MODEL workplace. Examination of the binding site topographies revealed pocket like surfaces for all of the modelled anti-hapten antibodies. The sizes and shapes of these pockets were influenced by the antigens they were selected against, with the main contributions to antigen binding coming from CDRs H2, H3, and L3 (Fig. 2). The significant influence of CDR H2 on antigen binding was *via* direct interaction with antigen and also *via* the less obvious indirect impact on the orientation and corresponding pocket shape and size delivered by the positioning of CDRs H3 and L3. In particular, two positions in CDR H2 (H58 and H59) greatly influenced antigen binding by controlling the pocket shape between CDR H2 and CDR L3 (Fig. 2). In all cases, position H59 was occupied with Phe, Tyr, Ile, or Leu, and these amino acids play a key role within a network or web of interactions with amino acids at positions H57, H58, H67, H69, L95a, and L95b (Fig. 3). These interactions enabled the remaining CDR H2 residues, H58 in particular, to be in direct contact with the different antigens. Analyses of position H58 revealed the presence of Phe in anti-SQA (A5, F9, and E10) clones (Fig. 3a), whilst Tyr was observed in anti-SQA (B3, F1, and E7) and all the anti-COP clones (Fig. 3a and c). For these clones at least, their binding sensitivities might indicate a preference for Phe when establishing interactions with hydrophobic targets like SQA and COP. In contrast, the presence of Tyr at this position appears to be important for the high binding sensitivity of clone POR B11, when compared to clones POR A7 and POR D11 that contained a H58 Phe (Fig. 3b). The four polar nitrogen atoms at the core of the POR structure should interact readily with the hydroxyl group of Tyr. For the anti-HSL antibodies, there was an Arg at position H58 in clones 2, 3, and 5, which contributed to binding of the polar lactone ring of HSL molecules. In contrast, the H58 Ile of clones 1, 4 and 6 has established hydrophobic interactions with the HSL molecules' aliphatic tail (Fig. 4a). Consequently, the role and influence of position H58 for antigen binding was confirmed and correlated with each hapten in terms of its chemical structure and polarity.

The surface topography of the area between CDRs H2 and H3 was also greatly influenced by a single position at H53 within CDR H2, which can establish interactions with H71, H52, and H54. The interaction of H53 and H71 was as predicted because all the post-selection clones contain CDR H2 with a class 1 conformation. For the less antigen-sensitive clones such as SQA F1, POR D11 and A7 (Fig. 3a and b), position H53 was occupied by an Arg. When the same position was represented by a smaller amino acid such as Ser (HSL 2–6, SQA A5 and SQA E10), Met (POR B11), or Thr (HSL 1), the clones all showed increased sensitivity to their respective target hapten. In general terms, this structural analysis suggested that the presence of relatively bulky amino acids (for example Arg) at position H53 might cause CDR H3 to be oriented further away from CDR H2 and towards CDR L2. This repositioning appears to partially occlude the hapten binding pocket (Fig. 3). Furthermore, we were able to explore this hypothesis further and show, using computer generated binding site “mutants” and their subsequent modelling, that changes in amino acid composition at position 53 was predicted to cause significant and functionally profound re-positioning of the CDR3 loop (results not shown).

### Electrostatic potential measurements of the post-selection clones

The surface-mapped electrostatic energy was examined across the entire population of post-selection clones. Here, four antibodies, one for each target, have been selected for illustration purposes (Fig. 4). SQA E10, POR B11, and COP H3 antibodies generally have relatively uncharged binding pockets ideally suited to their hydrophobic targets, whilst in stark contrast, the HSL antibodies have a positively charged binding pocket, as typified by the HSL 1 antibody. This positively charged binding pocket might be required to attract HSL molecules that are rich in oxygen molecules. These observations were supported by measurements of total electrostatic potential utilizing Adaptive Poisson-Boltzmann Solver (APBS) Version 0.5.1 in Python Molecule Viewer (PMV) 1.5.6. The predicted electrostatic energies of the anti-SQA antibodies ranged from 4.0 E + 04 (clones A5 and B3) to <3.8 E + 04 kJ/mol. Low electrostatic energy (<3.8 E + 04 kJ/mol) was also predicted for the three POR clones. In contrast, most of the anti-COP antibodies had a higher electrostatic energy (>4.2 E + 04 kJ/mol). The recorded energies of anti-HSL antibodies all had predicted values around 4.1 E + 04 kJ/mol. The total electrostatic potential of these antibodies complements the hydrophobic/hydrophilic nature of these haptens (Table 2).

## Discussion

Antibody fragments (scFv) are invaluable protein scaffolds that have been extensively used as diagnostics and more recently therapeutics [42]. They can recognize and bind to a diverse range of antigens including polypeptides, carbohydrates, lipids, nucleic acids, or even small hapten molecules. Haptens are not inherently immunogenic due to their small size but can elicit anti-hapten responses when coupled to a suitable immunogenic carrier protein prior to immunization. Sheep immunization followed by library construction and characterization by phage display technology has proved to be a successful strategy to develop highly sensitive anti-hapten antibodies [16–18].

The binding of antibody to antigen is principally generated by complementarities of the binding surfaces, which allows various interactions between the two molecules including the formation of hydrogen bonds, salt bridges, and van der Waals interactions [43]. This investigation of antibody-hapten interactions has therefore focused on hapten polarity and structural flexibility. The isolated SQA antibodies recognized the free SQA antigen in the micromolar (~2 μM) range. A possible reason for the moderate sensitivity to SQA might be attributed to its pronounced hydrophobic nature preventing/limiting the establishment of specific binding interactions, as has been reported for other hydrophobic targets [44]. Furthermore, binding sensitivities have been shown both experimentally [45] and computationally [46], to be enhanced to more rigid hapten structures. Whilst structural flexibility is clearly important, the POR used in this study has an unmetalated core, yet still generated antibodies with sub-micromolar sensitivity (POR B11, IC_50_ 270 nM). This contrasts with the moderate sensitivity to free antigen (micromolar range) observed for the cyclic aliphatic and nonpolar COP. Improvement in binding sensitivities have been reported in other studies following the chemical addition of polar OH or HO_3_SO groups at the carbon position 3 of COP (lithocholic acid or glycolithocholic acid sulfate) [47, 48]. It would appear therefore, that the presence of polar groups within the POR structure was more important in the generation of higher sensitivity binders than structural rigidity alone. When moderate flexibility and high polarity are combined (eg HSL antigens), super sensitive (picomolar range) binding interactions can be isolated. Here again polarity at the carbon 3 position (eg 3-OXO-C12-HSL) was a key determinant in overall sensitivity. One clear conclusion from this work is that the combination of relative structural rigidity with the presence of several polar groups is predictive of an enhanced hapten-antibody interaction.

This proposed influence of hapten chemistry on antibody binding might also be evident when examining amino acid distribution, CDRs lengths, and canonical classifications. Canonical combination 1–1 for CDR H1-H2 dominated both the pre and post-selection heavy chain sequences (Table 4). Structurally, CDR H1 bridges the two β-sheets and packs across the top of the VH domain. While class 1 of CDR H2 represents the shortest observed, and most commonly seen, loop for this CDR [49]. Almagro et al. [50] have reported the domination of these canonical classes within sheep heavy chain populations. In contrast to the restricted canonical classes of the sheep heavy chains, several canonical classes were observed in the pre-selection lambda chain sequences, with the class combination 6-1-X (CDRs L1-L2-L3) having the greatest representation in each of the three libraries. This newly proposed class (X) for CDR L3 (10 amino acids length) was found in 60-80 % of the pre-selection sequences, and interestingly in the less sensitive post-selection clones. The high incidence of canonical class (X) within the pre-selection clones, but not in the highly sensitive post-selection sequences, suggest this class is not pre-disposed to binding haptens as a group. CDR length comparisons revealed high conservation in CDRs H1, H2, and L2, but much more variability in CDRs L1, H3, and L3 (Table 3). Whereas the post-selection clones CDRs have shown high level of length conservation to accommodate each hapten. A CDR H3 with 12 amino acids was observed with SQA, POR, and HSL post-selection clones (Table 3). Anti-haptens antibodies with 12 amino acid CDR H3 have been reported previously against arsenate and phosphorylcholine [51], and it has been postulated that this is the minimum length to enable CDR H3 to form part of a pocket-like binding site. Interestingly, where CDR H3 lengths are 13 amino acids or greater, it has also been postulated that they become increasingly exposed to solvent, resulting in a more unstable structure and less defined binding site [52].

Antibody-antigen sensitivity is of course greatly influenced by the type of amino acids in contact with the different hapten targets. Analyses of the pre and post-selection sequences revealed more conserved sites in the post-selection sequences, when compared to their pre-selection counterparts, especially within the FW regions (Table 5). This type of conservation phenomenon has been reported previously but to a lesser extent (17 conserved sites), following examination of the 76 common core residues of ~5300 Kabat sequences of antibodies to a full range of different antigen classes [53]. One possible reason for the very high level of conservation we have seen is as an artefact of the antibody isolation process. Phage enrichment is known to be significantly influenced by toxicity and expressibility of the displayed antibody binding sites [54, 55]. However, the conserved positions seen here are consistent even across clones isolated from different libraries, or from structurally dissimilar haptens or different panning strategies. The only shared factor here is the overall low molecular weight of the haptens and the need to form a binding site shape able to accommodate them. These structural and topographical perquisites could drive high conservation in the FW regions, especially FW3, and result in the selection of clones from only a small subset of the full repertoire diversity.

Whilst all the homology-modelled sheep antibodies possess pocket-like binding sites with various shapes and sizes (Fig. 2), the development of more compact binding pockets has been attributed to closure of the VL-VH interface of the anti-hapten antibodies and improved binding sensitivities [28]. Here, the shape and overall size of the binding pockets was greatly influenced by a small number of amino acid positions in CDR H2 (H59 and H58, and H53) that appeared to control the orientation of CDRs H3 and L3. Position H59 was occupied with Phe, Tyr, Ile, or Leu, and was involved in a network of interactions with positions H57, H67, H69, L95a, and L95b. These interactions have enabled the remaining of CDR H2 amino acids, especially position H58, to be oriented toward the target antigens. Position H58 is known from previous work to have an important role in hapten recognition [20, 56]. The average relative side chain solvent-accessible surface of this position was estimated at 50-75 % [35]. This study is able to correlate the amino acids found at this site with antigen polarity, and with Phe, Tyr, and Arg pairing up with SQA and COP, POR, and HSL, respectively. Position H53 appears to impart a second and key structural influence on CDR H3 (Fig. 2 and 3). This residue is at the tip of CDR H2 and is believed to pack against residues at positions H71 and H29 [57]. From our models, the presence of Arg at this position causes steric hindrance with CDR H3 pushing this important CDR away from CDR H2 and towards CDR L2 (clones SQA F1 and POR D11 and A7). In concert, the residues found at these three positions in CDR H2 (H59, H58, and H53) appear to exert a significant influence over the size and shape of the hapten binding pockets.

Previous studies have concluded that antibody-protein interactions are based on "charge complementarity" and "electrostatic complementarity" [58], and that this complementarity is important in defining binding site specificity [59]. Therefore, it was interesting to examine whether this proposed electrostatic complementarty could be expanded to hapten antigens. Here, hapten complementarity is not limited to shape and size but extends also to surface electrostatic potential. SQA, POR, and COP binding pockets have hydrophobic surfaces, in complete contrast to the positively charged surfaces of anti-HSL antibodies (Fig. 4). These observations are further supported by the electrostatic optimization of anti-hapten antibodies specific for p-nitrophenyl phosphonate, fluorescein, and N-(P-cyanophenyl)-N-(diphenylmethyl)-guanidiniumacetic acid [60–62]. Consequently, we can predict that antibodies with high binding sensitivity show an improved capacity to recognize haptens by establishing electrostatically complementary binding pockets.

## Conclusion

Various structural and molecular factors appear to profoundly influence the successful binding of antibodies to hapten molecules. Haptens possessing a relatively rigid chemical backbone, together with the presence of polar groups, are much more likely to be recognized by antibodies with high sensitivity. These highly sensitive antibodies tend to show an improved capacity to recognize their antigens by establishing complementary binding pockets. These complementarities are influenced by amino acid composition and control the pocket size, shape, and electrostatic potential.

## Abbreviations

CDR: Complementarity determining regions
COP: Coprostane
Da: Dalton
FW: Framework
HSL: Homoserine lactone
POR: Etioporphyrin
scAb: Single chain antibody
scFv: Single-chain variable fragments
SQA: Squalane

## References

[1] Wu TT, Kabat EA (1970). An analysis of the sequences of the variable regions of bence jones proteins and myeloma light chains and their implications for antibody complementarity. J Exp Med.

[2] Padlan EA (1994). Anatomy of the antibody molecule. Mol Immunol.

[3] Bassing CH, Swat W, Alt FW (2002). The mechanism and regulation of chromosomal V(D)J recombination. Cell.

[4] Neuberger MS, Williams GT, Mitchell EB, Jouhal SS, Flanagan JG, Rabbitts TH (1985). A hapten-specific chimaeric IgE antibody with human physiological effector function. Nature.

[5] Weill J, Reynaud C (1996). Rearrangement/hypermutation/gene conversion: When, where and why?. Immunol Today.

[6] Stavnezer J, Guikema JEJ, Schrader CE (2008). Mechanism and regulation of class switch recombination. Annu Rev Immunol.

[7] Chothia C, Lesk AM (1987). Canonical structures for the hypervariable regions of immunoglobulins. J Mol Biol.

[8] Al-Lazikani B, Lesk AM, Chothia C (1997). Standard conformations for the canonical structures of immunoglobulins. J Mol Biol.

[9] Martin ACR, Thornton JM (1996). Structural families in loops of homologous proteins: Automatic classification, modelling and application to antibodies. J Mol Biol.

[10] Chothia C, Lesk AM, Gherardi E, Tomlinson IM, Walter G, Marks JD (1992). Structural repertoire of the human VH segments. J Mol Biol.

[11] Tomlinson LM, Cox JPL, Gherardi E, Lesk AM, Chothia C (1995). The structural repertoire of the human Vκ domain. EMBO J.

[12] Wu S, Cygler M (1993). Conformation of complementarity determining region L1 loop in murine IgG λ light chain extends the repertoire of canonical forms. J Mol Biol.

[13] Vargas-Madrazo E, Lara-Ochoa F, Almagro JC (1995). Canonical structure repertoire of the antigen-binding site of immunoglobulins suggests strong geometrical restrictions associated to the mechanism of immune recognition. J Mol Biol.

[14] Lara-Ochoa F, Almagro JC, Vargas-Madrazo E, Conrad M (1996). Antibody-antigen recognition: A canonical structure paradigm. J Mol Evol.

[15] Brichta J, Hnilova M, Viskovic T (2005). Generation of hapten-specific recombinant antibodies: Antibody phage display technology: A review. Vet Med.

[16] Palliyil S, Downham C, Broadbent I, Charlton K, Porter AJ (2014). High-sensitivity monoclonal antibodies specific for homoserine lactones protect mice from lethal pseudomonas aeruginosa infections. Appl Environ Microbiol.

[17] Charlton K, Harris WJ, Porter AJ (2001). The isolation of super-sensitive anti-hapten antibodies from combinatorial antibody libraries derived from sheep. Biosensors Bioelectron.

[18] Sathe M, Derveni M, Broadbent G, Bodlenner A, Charlton K, Ravi B (2011). Synthesis and characterisation of immunogens for the production of antibodies against small hydrophobic molecules with biosignature properties. Anal Chim Acta.

[19] Webster DM, Henry AH, Rees AR (1994). Antibody-antigen interactions. Curr Opin Struct Biol.

[20] MacCallum RM, Martin ACR, Thornton JM (1996). Antibody-antigen interactions: Contact analysis and binding site topography. J Mol Biol.

[21] Niemi MH, Takkinen K, Amundsen LK, Söderlund H, Rouvinen J, Höyhtyä M (2011). The testosterone binding mechanism of an antibody derived from a naïve human scFv library. J Mol Recognit.

[22] Peterson EC, Gunnell M, Che Y, Goforth RL, Carroll FI, Henry R (2007). Using hapten design to discover therapeutic monoclonal antibodies for treating methamphetamine abuse. J Pharmacol Exp Ther.

[23] Tabares-Da Rosa S, Rossotti M, Carleiza C, Carrión F, Pritsch O, Ahn KC (2011). Competitive selection from single domain antibody libraries allows isolation of high-affinity antihapten antibodies that are not favored in the llama immune response. Anal Chem.

[24] Li Y, Cockburn W, Kilpatrick JB, Whitelam GC (2000). High affinity ScFvs from a single rabbit immunized with multiple haptens. Biochem Biophys Res Commun.

[25] Ramirez-Benitez MDC, Almagro JC (2001). Analysis of antibodies of known structure suggests a lack of correspondence between the residues in contact with the antigen and those modified by somatic hypermutation. Proteins.

[26] Livesay DR, Subramaniam S (2004). Conserved sequence and structure association motifs in antibody-protein and antibody-hapten complexes. Protein Eng Design Selec.

[27] Collis AVJ, Brouwer AP, Martin ACR (2003). Analysis of the antigen combining site: Correlations between length and sequence composition of the hypervariable loops and the nature of the antigen. J Mol Biol.

[28] Pellequer J, Chen S-W, Roberts VA, Tainer JA, Getzoff ED (1999). Unraveling the effect of changes in conformation and compactness at the antibody V(L)-V(H) interface upon antigen binding. J Mol Recognit.

[29] Shaw I, O'Reilly A, Charleton M, Kane M (2008). Development of a high-affinity anti-domoic acid sheep scFv and its use in detection of the toxin in shellfish. Anal Chem.

[30] Charlton KA, Moyle S, Porter AJR, Harris WJ (2000). Analysis of the diversity of a sheep antibody repertoire as revealed from a bacteriophage display library. J Immunol.

[31] Charlton KA, Porter AJ (2002). Isolation of anti-hapten specific antibody fragments from combinatorial libraries. Methods Mol Biol.

[32] Hayhurst A, Harris WJ (1999). Escherichia coli skp chaperone coexpression improves solubility and phage display of single-chain antibody fragments. Protein Expr Purif.

[33] Strachan G, Grant SD, Learmonth D, Longstaff M, Porter AJ, Harris WJ (1998). Binding characteristics of anti-atrazine monoclonal antibodies and their fragments synthesised in bacteria and plants. Biosensors Bioelectron.

[34] Grant SD, Porter AJ, Harris WJ (1999). Comparative sensitivity of immunoassays for haptens using monomeric and dimeric antibody fragments. J Agric Food Chem.

[35] Honegger A, Plückthun A (2001). Yet another numbering scheme for immunoglobulin variable domains: An automatic modeling and analysis tool. J Mol Biol.

[36] Ewert S, Honegger A, Plückthun A (2004). Stability improvement of antibodies for extracellular and intracellular applications: CDR grafting to stable frameworks and structure-based framework engineering. Methods.

[37] Kabat E, Wu T, Perry H, Gottesman K, Foeller C (1991). Sequences of proteins of immunological interest.

[38] Schwede T, Kopp J, Guex N, Peitsch MC (2003). SWISS-MODEL: An automated protein homology-modeling server. Nucleic Acids Res.

[39] Bordoli L, Kiefer F, Arnold K, Benkert P, Battey J, Schwede T (2009). Protein structure homology modeling using SWISS-MODEL workspace. Nat Protoc.

[40] Trott O, Olson AJ (2010). Software news and update AutoDock vina: Improving the speed and accuracy of docking with a new scoring function, efficient optimization, and multithreading. J Comput Chem.

[41] Steipe B, Pluckthun A, Huber R (1992). Refined crystal structure of a recombinant immunoglobulin domain and a complementarity-determining region 1-grafted mutant. J Mol Biol.

[42] Weisser NE, Hall JC (2009). Applications of single-chain variable fragment antibodies in therapeutics and diagnostics. Biotechnol Adv.

[43] Yokota A, Tsumoto K, Shiroishi M, Kondo H, Kumagai I (2003). The role of hydrogen bonding via interfacial water molecules in antigen-antibody complexation: The HyHEL-10-HEL interaction. J Biol Chem.

[44] James LC, Tawfik DS (2003). The specificity of cross-reactivity: Promiscuous antibody binding involves specific hydrogen bonds rather than nonspecific hydrophobic stickiness. Protein Sci.

[45] Zimmermann J, Romesberg FE, Brooks CL, Thorpe IF (2010). Molecular description of flexibility in an antibody combining site. J Phys Chem B.

[46] Thorpe IF, Brooks CL (2007). Molecular evolution of affinity and flexibility in the immune system. Proc Natl Acad Sci U S A.

[47] Ikegawa S, Yamamoto T, Miyashita T, Okihara R, Ishiwata S, Sakai T (2008). Production and characterization of a monoclonal antibody to capture proteins tagged with lithocholic acid. Anal Sci.

[48] Kobayashi N, Katsumata H, Uto Y, Goto J, Niwa T, Kobayashi K (2002). A monoclonal antibody-based enzyme-linked immunosorbent assay of glycolithocholic acid sulfate in human urine for liver function test. Steroids.

[49] Efimov AV (1993). Patterns of loop regions in proteins. Curr Opin Struct Biol.

[50] Almagro JC, Martinez L, Smith SL, Alagon A, Estevez J, Paniagua J (2006). Analysis of the horse VH repertoire and comparison with the human IGHV germline genes, and sheep, cattle and pig VH sequences. Mol Immunol.

[51] Johnson G, Wu TT (1998). Preferred CDRH3 lengths for antibodies with defined specificities. Int Immunol.

[52] Kuroda D, Shirai H, Kobori M, Nakamura H (2008). Structural classification of CDR-H3 revisited: A lesson in antibody modeling. Proteins.

[53] Chothia C, Gelfand I, Kister A (1998). Structural determinants in the sequences of immunoglobulin variable domain. J Mol Biol.

[54] Ravn U, Gueneau F, Baerlocher L, Osteras M, Desmurs M, Malinge P, et al. By-passing in vitro screening - next generation sequencing technologies applied to antibody display and in silico candidate selection. Nucleic Acids Res. 2010;38. 10.1093/nar/gkq789 PMC2995085 20846958

[55] Scott N, Reynolds CB, Wright MJ, Qazi O, Fairweather N, Deonarain MP. Single-chain fv phage display propensity exhibits strong positive correlation with overall expression levels. BMC Biotechnol. 2008;8. 10.1186/1472-6750-8-97 PMC2630973 19113995

[56] Honegger A, Spinelli S, Cambillau C, Plückthun A (2005). A mutation designed to alter crystal packing permits structural analysis of a tight-binding fluorescein-scFv complex. Protein Sci.

[57] Tramontano A, Chothia C, Lesk AM (1990). Framework residue 71 is a major determinant of the position and conformation of the second hypervariable region in the VH domains of immunoglobulins. J Mol Biol.

[58] Braden BC, Poljak RJ (1995). Structural features of the reactions between antibodies and protein antigens. FASEB J.

[59] McCoy AJ, Chandana Epa V, Colman PM (1997). Electrostatic complementarity at protein/protein interfaces. J Mol Biol.

[60] Chong LT, Duan Y, Wang L, Massova I, Kollman PA (1999). Molecular dynamics and free-energy calculations applied to affinity maturation in antibody 48G7. Proc Natl Acad Sci U S A.

[61] Lippow SM, Wittrup KD, Tidor B (2007). Computational design of antibody-affinity improvement beyond in vivo maturation. Nat Biotechnol.

[62] Livesay D, Linthicum S, Subramaniam S (1999). pH dependence of antibody: Hapten association. Mol Immunol.

[63] Chothia C, Lesk AM, Tramontano A, Levitt M, Smith-Gill SJ, Air G (1989). Conformations of immunoglobulin hypervariable regions. Nature.

